# A Complicated Case of Triple Valve Infective Endocarditis in an IV Drug User with a Bicuspid Aortic Valve Requiring Three Separate Salvage Operations: A Case Report and Literature Review

**DOI:** 10.1155/2015/291079

**Published:** 2015-06-03

**Authors:** Shahzad Khan, Athanasios Smyrlis, Dmitry Yaranov, David Oelberg, Eric Jimenez

**Affiliations:** Danbury Hospital, Western Connecticut Health Network, 187 Willow Springs, New Milford, CT 06776, USA

## Abstract

Infective endocarditis (IE) is an infection of the endocardium that involves valves and adjacent mural endocardium or a septal defect. Local complications include severe valvular insufficiency, which may lead to intractable congestive heart failure and myocardial abscesses. If left untreated, IE is generally fatal. Diagnosing IE can be straightforward in patients with the typical oslerian manifestations such as bacteremia, evidence of active valvulitis, peripheral emboli, and immunologic vascular phenomena. In the acute course, however, the classic peripheral stigmata may be few or absent, particularly among intravenous drug abuse (IVDA) patients in whom IE is often due to a* S. aureus *infection of right-sided heart valves. We present a complicated case of a very aggressive native aortic valve* MSSA* (*methicillin sensitive Staphylococcus aureus*) IE in a young adult male with a past medical history of bicuspid aortic valve and IV drug abuse. His clinical course was complicated by aortic valve destruction and development of third-degree AV block, as well as an aorto-left atrial fistula requiring emergent operation for AV replacement and patch repair. The patient required two reoperations for recurrent endocarditis and its complications.

## 1. Background

Infective endocarditis (IE) is an infection of the endocardium that could involve or affect the valves and adjacent structures of the heart. IE can be caused by a wide variety of microorganisms fungal or bacteria (*Streptococcus viridans*,* Streptococcus gallolyticus*,* Staphylococcus aureus*,* HACEK* group). Although not common, it can be a fatal pathological condition if not identified and treated especially in those that are older and have congenital or valvular heart defects or other comorbidities that delay or impede the healing process. Different reports indicate that, in the United States, there may be up 15,000 new cases of IE reported every year [1].

High risk patient groups for developing IE include those with a history of IV drug use, congenital heart disease, increased age, prosthetic heart valves, and end stage renal disease on hemodialysis [2].

5% to 15% of hospital admissions of IV drug users (IDU) are attributed to IE [2]. Female gender and HIV have been identified as risk factors among IDU. Intravenous drug use increased chances of getting IE similar to those found in the prosthetic heart valve population, around 1% per year [3].

Bicuspid aortic valve is the most common congenital malformation. It affects 1-2% of the general population and has a 3 : 1 predilection of males to females and is a well-described risk factor for IE [4]. Lamas and Eykyn demonstrated that 7–25% of the IE population had a past medical history of a congenital bicuspid aortic valve. They also found that these patients are more likely to have a worse prognosis such as developing heart failure, valvular destruction, and/or perivalvular/myocardial abscess [5]. Abscess formation in the aortic annulus can be complicated by complete heart block or bundle branch block in up to 45% of cases.

## 2. Case Presentation

The patient is a 36-year-old male with bicuspid aortic valve and history of IV drug use (which he initially denied). He presented to another hospital approximately one week after developing a diffuse rash, generalized muscle aches, profound weakness, fevers, and chills. At that time, he was diagnosed with an upper respiratory infection and was discharged from the emergency room on oral antibiotics. After five days he returned back to the same hospital with complaints of persistent high fevers and was admitted. During his stay, he was found to have 4/4 positive blood cultures prompting a transfer to our hospital for further evaluation and management.

**Figure 1:**
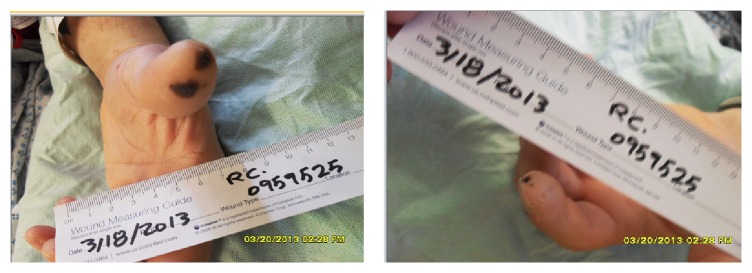
Vasculitic lesions. Multiple embolic lesions were present on the left fourth and fifth fingertips and left first toe.

His physical exam on presentation was significant for Janeway lesions as well as multiple vasculitic lesions on his fingers and toes (Figure 1) as well as a grade II/IV diastolic murmur. IE was suspected. Blood cultures were obtained and he was promptly started on intravenous Ceftriaxone and Vancomycin. At the time of admission the patient stated that he had a peripherally inserted central catheter placed three months before for treatment of non-Hodgkin's lymphoma at a neighboring hospital that was removed six days prior to presentation. He later admitted to an ICU nurse that the IV line had been inserted by a friend in a hotel room for intravenous injections of illicit drugs.

**Figure 2:**
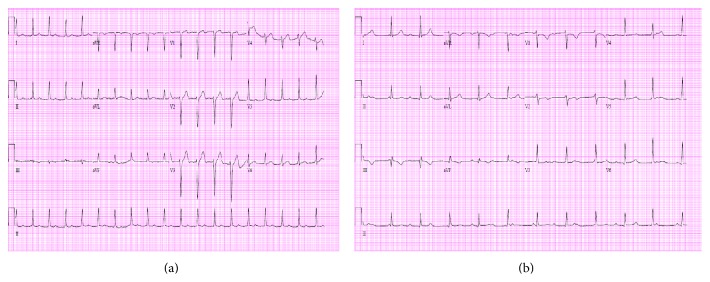
Electrocardiogram. Day 1 ECG on left and day 2 on right. Day 1 ECG reveals probable sinus tachycardia with first-degree heart block at 111 bpm. Day 2 ECG shows complete heart block with a ventricular rate of 67 bpm.

**Figure 3:**
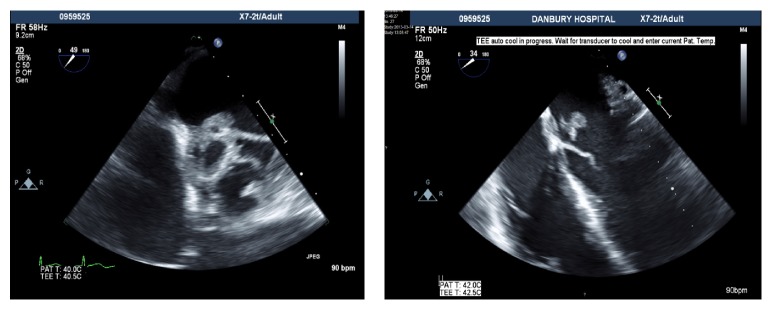
Transesophageal echocardiogram. Mass seen on anterior leaflet measuring 1.2 × 1.8 cm with perforation of the leaflet. Bicuspid aortic valve with severe aortic regurgitation and abscess cavity and fistula extending from right coronary cusp to left atrium.

Blood cultures from the previous hospital grew* MSSA*. Repeat bacterial and fungal cultures at our institution came back negative. Initial ECG showed probable sinus tachycardia with first-degree atrioventricular block (Figure 2). Transesophageal echocardiogram (TEE) demonstrated a bicuspid aortic valve with multiple mobile echo densities along both the anterior and posterior leaflets with the largest one measuring 1.8 cm (Figure 3).

Overnight the patient was noted to have arrhythmias on telemetry and a repeat ECG showed new complete heart block (Figure 2). The patient was then taken to the operating room (OR) emergently where he had an aortic valve replacement with bovine pericardial prosthesis, pericardial patch repair of the aorto-left atrial fistula with a St. Jude Medical bovine pericardial patch, and removal of infected debris from his tricuspid valve and mitral valve. Intraoperative histopathological results of the aortic valve indicated marked acute inflammation, fibroinflammatory debris, coccoid bacteria, and calcifications. The tricuspid valve was also involved with fibroinflammatory debris consistent with endocarditis. Cultures of the specimens grew out* MSSA* and antibiotics were changed to Cefazolin. The patient had an uncomplicated postoperative course and was discharged one week later with a plan of a six-week course of intravenous Cefazolin and close outpatient follow-up.

The patient missed his initial two follow-ups with his infectious disease (ID) doctors and there was concern for recurrent IVDA which he denied. After multiple attempts to reach him he started to follow up and became routine in his treatment. In the outpatient setting he also had repeat blood cultures taken at 3 weeks which came back negative. Six weeks after discharge he complained to his cardiologist of night sweats, fatigue, and shortness of breath. A repeat transthoracic echocardiogram was performed showing new vegetations over the bioprosthetic aortic valve with an abscess cavity and fistulization into the right atrium, moderate/severe tricuspid insufficiency, presence of ventricular septal defects, and new vegetations in the mitral valve. He was started empirically on Cefazolin with a continuous antimicrobial infusion to maintain adequate minimum inhibitory concentration and Rifampin. Repeat blood cultures (bacterial and fungal) were negative. He was taken back to the operating room where he had a redo aortic valve replacement with a Carpentier-Edwards ThermaFix bovine pericardial bioprosthesis, closure of the VSD, and subaortic fistula into the right atrium and into the left atrium using a St. Jude Medical bovine pericardial patch. The aortic bioprosthetic valve grossly was a tricuspid porcine valve measuring 3.7 cm (diameter) × 1.5 cm (thickness) with a tan white smooth surface with green sutures attached. Histopathological examination of the valve and fistula showed fibroconnective tissue with associated foci of inflammatory exudates, cultures and staining of which were negative for bacteria and fungi. He was eventually discharged on oral Ciprofloxacin and Rifampin for a total 6-week course.

One week after discharge he developed symptoms of heart failure including shortness of breath and nocturnal dyspnea. He had a repeat echocardiogram that showed a prosthetic valve vegetation with a large aneurysmal membrane between the prosthesis and mitral annulus and evidence of left ventricular outflow tract left atrial fistula with severe regurgitation. A ventricular septal defect was present with left ventricular outflow tract right atrial flow and another vegetation was seen over the anterior mitral valve. Blood cultures (bacterial and fungal) were taken at the time both of which were negative and he was empirically started on Vancomycin, Gentamycin, Cefepime, Rifampin, and Micafungin. The patient was then taken back to the OR for the final time where he had a St. Jude mechanical aortic valve replacement with debridement of the other valves. Intraoperative histological analysis showed fibroconnective tissue that was negative for bacterial and fungal staining and cultures as well as negative for 16sRNA and 18sRNA. The patient was then stabilized and discharged on Rifampin plus Vancomycin for a total course of 6 weeks and Gentamicin for 2 weeks. Upon discharge he followed with his doctors regularly and did not have any further cardiac complications.

## 3. Discussion

This was a highly complex case due to the severe pathology with multiple valve involvement as well as the patients' social and behavioral issues. His management was particularly challenging as he initially failed to present to the infusion center and antibiotic course was interrupted while he was likely using the IV access for IVDU. The patient did not comply with outpatient follow-up consistently and he was initially not willing to take Warfarin; thus a mechanical valve was not an option.

The second time he presented, it was assumed that he had a persistent* MSSA* infection versus reinfection; therefore he was empirically started on Cefazolin with Rifampin and discharged on oral Ciprofloxacin plus Rifampin once cultures came back negative. Although persistent infection was the most likely culprit, this antibiotic regimen minus the Gentamycin lacked the synergistic strength that may have been required to kill his persistent infection. Also although Ciprofloxacin does provide gram-negative coverage, it is not the best choice if* Pseudomonas aeruginosa *is in the pathogen differential. Baddour et al. demonstrated that although not common, 95% of those infected with* P. aeruginosa* had a positive history of IVDU. Another difficult choice was to discharge the patient on oral antibiotics due to concern of recurrent IVDU through the peripherally inserted central catheter. This was a conscious decision which took into account his history and lack of initial follow-up. It is not necessarily the preferred method but Ciprofloxacin and Rifampin have been shown to achieve good bioavailability in the oral form [6]. Ultimately he came back for a third time and was initially treated with Cefepime, Vancomycin, Gentamycin, Rifampin, and Micafungin. His cultures came back negative and he received a 2-week course of Gentamycin with a 6-week course of Vancomycin with Rifampin. He was subsequently compliant with all his follow-ups and treatment recommendations. One year after the last surgery he remains free of endocarditis and he has been able to abstain from illegal drug use.

The social aspects and intricacies of treating IV drug users for endocarditis result often in a difficult and unstable physician-patient relationship. Creating rapport with such patients requires a significant investment of time and energy and it is absolutely essential for treatment success.
